# Looking Back to Move Forward: Lessons Learned from a Successful, Sustainable, Replicable Model of Adolescent and Young Adult Program of a Tertiary Cancer Care Center

**DOI:** 10.1089/jayao.2021.0156

**Published:** 2022-04-19

**Authors:** Natalie Pitch, Stephanie Stefaniuk, Meghan MacMillan, Jennifer Catsburg, Abha A. Gupta, Tushar Vora

**Affiliations:** ^1^ Temerty Faculty of Medicine, University of Toronto, Toronto, Canada.; ^2^ Adolescent and Young Adult Program, Department of Supportive Care, Princess Margaret Cancer Centre, Toronto, Canada.; ^3^ Alberta Health Services, Edmonton, Canada.; ^4^ Division of Hematology/Oncology, Hospital for Sick Children, University of Toronto, Toronto, Canada.

**Keywords:** adolescent and young adult, program analysis, survivorship, AYA concerns

## Abstract

**Background:**

The Princess Margaret Cancer Centre (PM) established the adolescent and young adult (AYA) oncology program in 2014 to address the unique needs of AYA by delivering targeted, evidence-based care through a multidisciplinary team.

**Methods:**

We performed a retrospective analysis of patients who underwent a consultation with the PM AYA program from 2014 to 2020. The association between the health domain concerns reported and age at consultation, cancer diagnoses, and time since diagnosis was analyzed using chi-square test of independence in SPSS.

**Results:**

In our cohort of 1128 AYA, the median age at assessment was 28.2 years. The most common diagnoses were lymphoma (*n* = 251, 22.2%), leukemia (*n* = 207, 18.4%), and breast cancer (*n* = 162, 14.4%). The most common concerns reported were related to fertility (*n* = 882, 78.2%) and work/school (*n* = 472, 41.8%). Fertility concerns were most common in 25–34 age group (443/540, 82.0%) and work-/school-related concerns were highest in 18–24 age group (191/355, 53.8%). Diagnoses significantly affect majority of concerns reported. Fertility concerns were most common in AYA consulted near diagnosis, while body image-, exercise-, and diet-related concerns were more frequently reported, while on active treatments.

**Conclusions:**

Supporting fertility concerns remains the cornerstone of any successful AYA program. Work-/school-related concerns deserve more elucidation and attention. We identified important patterns in the health-related concerns of AYA, especially as they relate to age, diagnoses, and time since diagnosis. This insight will guide us for improving patient-centered care delivery to AYA.

## Introduction

Adolescents and young adults (AYAs) diagnosed with cancer face unique set of challenges, given their dynamic developmental stage. During this transitional period, AYA work toward establishing autonomy, developing a positive body image and sexual identity, fostering peer relationships, starting careers, and beginning families.^1–3^ Receiving a diagnosis of cancer and undergoing associated treatments can disrupt the achievements of these key milestones. Accordingly, this population has psychosocial and supportive care needs that are especially complex and differentiate them from their pediatric and adult counterparts, and are thus not ideally suited for either pediatric or adult cancer care programs.^4,5^

Across diagnoses, AYA with cancer experience many unique challenges associated with their diagnosis and subsequent therapies, such as undergoing fertility preservation, maintaining sexual health and body image, developing coping mechanisms, keeping a healthy diet, having financial security, and sustaining work/school commitments.^6–10^ AYA with cancer exhibit significantly poorer health-related quality of life relative to healthy populations.^11^ Comprehensive programs to address their unique needs have been attempted in various parts of the world by different groups such as Teenage Cancer Trust UK^12^ and The Australian Youth Cancer Service.^13^

Princess Margaret Cancer Centre (PM), the largest tertiary cancer referral center in Toronto, Canada, established its AYA Program in 2014 with the goal of delivering high-quality, evidence-based, comprehensive, and age-appropriate care to AYA. Over the last 7 years, we have aimed to deliver personalized care to AYA across their treatment journeys through a multidisciplinary team involving members from medical oncology, nursing, physiotherapy, psychology, psychiatry, social work, fertility specialists, occupational therapy, palliative care, physiatry, kinesiology, nutrition therapy, and community partners. Patient-reported value addition in the core domains of sexual health, fertility, social support, physical appearance, and work/school concerns, by the program, have been documented in the past.^14^

Now we report a retrospective audit of all patients registered in the PM AYA program from its inception to December 31, 2020, with aim to explore possible associations between the AYA health concerns reported and patient demographics. This would help improve our delivery of care and provide an example for other similar programs worldwide.

## Methods

### Patient inclusions

AYA are eligible for the program either through self-referral or by a health care provider, at any time during their cancer journey. This retrospective audit is limited to AYA between 18 and 39 years of age. Since PM is an adult cancer referral center, the lower age limit was 18 years. The program accepted referrals from patients with AYA-related health concerns outside this age range, but they have not been included in this analysis to maintain an AYA perspective.

### AYA consultations

The initial 1-hour consultation is led by a central nervous system (CNS) who uses a specially modified Edmonton symptom assessment scale to identify and triage the concerns reported in the following domains: fertility, sexual health, coping, body image, exercise, diet, work/school, finances, and social support (Table 1).^15^ The concerns reported were identified as being present or absent, irrespective of their intensity of grading on the scale (Yes—1–10, No—0). The concerns were reported in respect to time of consultation and not over a period of time. As a part of the initial intake, the CNS provides education that is reinforced with informational resources, especially to address concerns related to fertility preservation, peer support, and community resources.

**Table 1.** Modified Edmonton Symptom Assessment Screening Scale—Princess Margaret Cancer Centre, Adolescent and Young Adult Program

AYA screening tool												
Please circle the number that best describes you:												
No concern with work/school	0	1	2	3	4	5	6	7	8	9	10	Significant work/school concerns
No concern with finances	0	1	2	3	4	5	6	7	8	9	10	Significant financial concerns
Not anxious about my future	0	1	2	3	4	5	6	7	8	9	10	Very anxious about my future
I have excellent social supports	0	1	2	3	4	5	6	7	8	9	10	I do not have individuals in my life that are supporting me
No concern about my appearance	0	1	2	3	4	5	6	7	8	9	10	Significant concern about my appearance
No concerns about my sexual health	0	1	2	3	4	5	6	7	8	9	10	Significant concerns about my sexual health
No concerns about my fertility	0	1	2	3	4	5	6	7	8	9	10	Significant concerns about my fertility
No difficulty understanding info about my cancer	0	1	2	3	4	5	6	7	8	9	10	Significant difficulty understanding info about my cancer
No concerns with diet/nutrition	0	1	2	3	4	5	6	7	8	9	10	Significant concern with diet/nutrition
No difficulty navigating the hospital system	0	1	2	3	4	5	6	7	8	9	10	Significant difficulty navigating the hospital system
No concerns with physical activity/exercise	0	1	2	3	4	5	6	7	8	9	10	Significant concerns with physical activity/exercise

Depending on specific concerns voiced and addressed during this initial consultation, the CNS may then refer the patient to additional specialized care services. These services include fertility specialists, occupational and physical therapists, dieticians, social workers, psychologists, and psychiatrists. The CNS may also provide links to community organizations that provide additional support such as exercise and nutrition classes, mindfulness coaching, and workshops on topics such as return to work (RTW)/school, sexual health, body image, financial management, and peer-support groups. The CNS then records the details of consultation on AYA e-cancer registry. This objective method of consultation has been kept consistent since the inception of the program.

Before the coronavirus disease 2019 (COVID-19) pandemic, most of the consultations were in person at PM; however, telephonic and videoconferencing tools were introduced in March 2020 in response to pandemic protocols implemented by the hospital. Currently, consultations are either offered in person or virtually depending on patient preference and needs.

Patients are also given the opportunity to sign up for the AYA program's monthly newsletter to remain updated on services or activities delivered by the program. Follow-up appointments are offered based on need and AYA can reconnect with the program at any point throughout treatment and survivorship. Beyond patient care, the program also provides education to health care providers on AYA-specific issues through rounds, lectures, awareness events, and dissemination of research.

### Retrospective review

A retrospective review of all initial consultations of the PM AYA program was done from inception to December 31, 2020. Patient-related variables (age, disease type, and time since diagnosis) and health-related concerns reported during the initial consultations were retrieved from the AYA e-cancer care registry. AYA were grouped into three age categories (18–24, 25–34, and 35–39 years) at the time of assessment in the PM AYA program to understand possible associations of age with unique concerns.

The age categories roughly defined the three phases of AYA journey through adolescence, young adulthood, and late-young adulthood and their anticipatory different life stages and goals corresponding to education, career, and family.^16^ The time since diagnosis to initial assessment was categorized as 0–30 days, 31–90 days, 91–365 days, and after 365 days of date of diagnosis. The time since diagnosis to the date of consultation was categorized thus to reflect the cancer journeys of AYA through at diagnosis, starting on treatments, during treatments, and post-treatment, assuming that most cancer treatments would be completed by 1 year since diagnosis.

The associations between reported health concerns with age category, cancer diagnoses, and time since diagnosis were explored using chi-square test of independence in Statistical Package for Social Sciences (SPSS/PC; SPSS-25.0, Chicago). *p*-Value <0.05 was considered significant. We acknowledge the confounding effect of dependent variables and lack of further subgroup analysis to show individual significance as limitations of our study. However, the insights gained from this study will establish the foundation for further analysis and guide the improvement of PM AYA program care delivery.

This retrospective study was approved by the Quality Improvement Committee of University Health Network, Toronto, Canada.

## Results

### Demographics

From inception in 2014, until December 31, 2020, 1128 AYA have benefited from the services of the PM AYA program. The median age at assessment of this cohort was 28.2 years. There were 56 referrals outside of the 18–39 year range that the program honored, but they have not been included in this analysis. Table 2 shows the distribution of AYA across age groups, diagnoses, and time since diagnosis. Table 3 shows the statistical associations of health concern domains reported by our patient cohort with age at assessment, diagnoses, and time since diagnosis.

**Table 2.** Distribution of Adolescent and Young Adult Across Age at Assessment, Diagnoses, and Time since Diagnosis

	*n* (%)
Age at assessment (years), *n* = 1128 (%) (Range 18–39)	
18–24	355 (31.5)
25–34	540 (47.9)
35–39	233 (20.6)
Diagnoses, *n* = 1128 (%)	
Lymphoma	251 (22.2)
Leukemia	207 (18.4)
Breast	162 (14.4)
GU	102 (9.0)
Sarcoma	84 (7.4)
Gynecological	64 (5.7)
CNS	60 (5.3)
GI	52 (4.6)
H&N	36 (3.2)
Benign	29 (2.6)
BMT	24 (2.1)
Others	57 (5.1)
Time since diagnosis (days), *n* = 943 (%)	
0–30	305 (32.3)
31–90	242 (25.7)
91–365	167 (17.7)
>365	229 (24.3)

BMT, bone marrow transplant; CNS, central nervous system; GI, gastrointestinal; GU, genitourinary; H&N, head and neck.

**Table 3.** Reported Concerns Across Age at Assessment, Diagnoses and Time Since Diagnosis

	Fertility	Sexual health	Coping	Body image	Exercise	Diet	Financial	Work/school	Social
Total									
*n* (%)	882 (78.2)	224 (19.9)	283 (25.0)	164 (14.5)	267 (23.7)	221 (19.6)	92 (8.2)	472 (41.8)	61 (5.4)
Age at assessment (years) *n* = 1128									
18–24 (355, 31.5)	266 (74.9)	74 (20.8)	78 (22.0)	51 (14.4)	79 (22.3)	54 (15.2)	35 (9.9)	191 (53.8)	17 (4.8)
25–34 (540, 47.9)	443 (82.0)	104 (19.3)	144 (26.7)	78 (14.4)	127 (23.5)	112 (20.7)	47 (8.7)	201 (37.2)	30 (5.6)
35–39 (233, 20.7)	173 (74.2)	46 (19.7)	61 (26.2)	35 (15.0)	61 (26.2)	55 (23.6)	10 (4.3)	80 (34.3)	14 (6.0)
*p* value	0.011	0.843	0.259	0.972	0.545	0.044	0.044	<0.001	0.797
Diagnoses *n* = 1128									
Lymphoma (251, 22.2)	210 (83.7)	53 (21.1)	71 (28.3)	41 (16.3)	56 (22.3)	47 (18.7)	20 (8.0)	116 (46.2)	13 (5.2)
Leukemia (207, 18.4)	185 (89.4)	48 (23.2)	38 (18.4)	27 (13.0)	52 (25.1)	29 (14.0)	25 (12.1)	94 (45.4)	5 (2.4)
Breast (162, 14.4)	143 (88.3)	44 (27.2)	44 (27.2)	51 (31.5)	48 (29.6)	50 (30.9)	15 (9.3)	75 (46.3)	17 (10.5)
GU (102, 9.0)	82 (80.4)	9 (8.8)	15 (14.7)	8 (7.8)	19 (18.6)	18 (17.6)	5 (4.9)	31 (30.4)	3 (2.9)^a^
Sarcoma (84, 7.4)	45 (53.6)	14 (16.7)	25 (29.8)	9 (10.7)	27 (32.1)	15 (17.9)	6 (7.1)	43 (51.2)	3 (3.6)^a^
Gynecological (64, 5.7)	37 (57.8)	21 (32.8)	28 (43.8)	7 (10.9)	22 (34.4)	18 (28.1)	2 (3.1)^a^	30 (46.9)	5 (7.8)^a^
CNS (60, 5.3)	40 (66.7)	9 (15.0)	21 (35.0)	4 (6.7)^a^	12 (20.0)	15 (25.0)	2 (3.3)^a^	23 (38.3)	1 (1.7)^a^
GI (52, 4.6)	37 (71.2)	6 (11.5)	8 (15.4)	2 (3.8)^a^	8 (15.4)	4 (7.7)^a^	4 (7.7)^a^	14 (26.9)	4 (7.7)^a^
H&N (36, 3.2)	26 (72.2)	8 (22.2)	9 (25.0)	5 (13.9)	10 (27.8)	9 (25.0)	5 (13.9)	14 (38.9)	4 (11.1)^a^
Benign (29, 2.6)	21 (72.4)	2 (6.9)^a^	3 (10.3)^a^	2 (6.9)^a^	2 (6.9)^a^	4 (13.8)^a^	4 (13.8)^a^	8 (27.6)	0^a^
BMT (24, 2.1)	21 (87.5)	5 (20.8)	7 (29.2)	4 (16.7)^a^	1 (4.2)^a^	1 (4.2)^a^	0^a^	9 (37.5)	1 (4.2)^a^
Others (57, 5.1)	35 (61.4)	5 (8.8)	14 (24.6)	4 (7.0)^a^	10 (17.5)	11 (19.3)	4 (7.0)^a^	15 (26.3)	5 (8.8)
*p* value	<0.001	<0.001	<0.001	<0.001	0.006	<0.001	0.175	0.005	0.026
Time since diagnosis (days) *n* = 943									
0–30 (305, 32.3)	283 (92.8)	61 (20.0)	64 (21.0)	46 (15.1)	70 (23.0)	62 (20.3)	20 (6.6)	119 (39.0)	22 (7.2)
31–90 (242, 25.7)	209 (86.4)	52 (21.5)	71 (29.3)	43 (17.8)	82 (33.9)	67 (27.7)	22 (9.1)	116 (47.9)	10 (4.1)
91–365 (167, 17.7)	122 (73.0)	37 (22.2)	42 (25.1)	36 (21.6)	45 (26.9)	35 (21.0)	17 (10.2)	72 (43.1)	2 (1.2)
>365 (229, 24.3)	144 (62.9)	46 (20.1)	66 (28.8)	21 (9.2)	50 (21.8)	33 (14.4)	16 (7.0)	114 (49.8)	4 (1.7)
*p* value	<0.001	0.931	0.093	0.005	0.010	0.028	0.447	0.056	0.002

^a^ Not applicable—Expected cell size as per analysis ≥5, hence not applicable.

BMT, bone marrow transplant; CNS, central nervous system; GI, gastrointestinal; GU, genitourinary; H&N, head and neck.

The majority of AYA were in the 25–34 age group (540, 47.9%). Almost one-third (355, 31.5%) of the cohort were in the 18–24 age group and the remainder (233, 20.7%) were in the 35–39 age group. The most common diagnoses were lymphoma (251, 22.2%), leukemia (207, 18.4%), breast cancer (162, 14.4%), genitourinary cancer (102, 9.0%), and sarcoma (84, 7.4%). The sample size used to evaluate time since diagnosis was smaller (*n* = 943) because patients without a documented date of diagnosis were excluded from this analysis. The greatest proportion of patients were consulted within 30 days of their diagnosis (305, 32.3%), while 229 (24.3%) patients were consulted more than a year after diagnosis.

Among the entire cohort, the most common concern reported was fertility (882, 78.2%) and the second most common concern was related to work/school (472, 41.8%). The remaining concerns reported in decreasing frequency were coping (283, 25.0%), exercise (267, 23.7%), sexual health (224, 19.9%), diet (221, 19.6%), body image (164, 14.5%), financial (92, 8.2%), and social support (61, 5.4%).

### Impact of age at assessment

The two most common concerns reported (fertility and work/school) did significantly differ by age at assessment. Fertility concerns were most common in 25–34 age group (443/540, 82.0%) compared to 18–24 age group (266/355, 74.9%) and the 35–39 age group (173/233, 74.2%), with *p* = 0.011. The proportion of work-/school-related concerns was highest in the 18–24 age group (191/355, 53.8%) compared to the 25–34 age group (201/540, 37.2%) and the 35–39 age group (80/233, 34.2%), with *p* < 0.001. There was also a significant association between financial concerns and age group, with the highest proportion of financial concerns in the 18–24 age group, with *p* = 0.044. The remaining domains of concerns were not related to age at assessment.

### Impact of diagnoses

Our analysis shows that diagnoses affect the concerns reported across all domains, except those related to finance, with the majority of concerns being significantly affected (*p* < 0.001). This highlights the need for personalized approaches for care delivery to AYA based on their diagnoses.

The highest proportion of patients with fertility concerns were in the leukemia cohort (185/207, 89.4%), followed by breast cancer (143/162, 88.3%), lymphoma (210/251, 83.7%), and genitourinary cancer (82/102, 80.4%). Importantly, the lowest proportion of patients reporting fertility concerns (sarcoma, 45/84, 53.6%) was also substantial. The proportion of work-/school-related concerns was highest in AYA with sarcoma (43/84, 51.2%) followed by patients with gynecological cancers (30/64, 46.9%), breast cancer (75/162, 46.3%), lymphoma (116/251, 46.2%), and leukemia (94/207, 45.4%). As expected, the proportion of sexual health concerns was greatest in AYA with gynecological cancers (21/64, 32.8%) and breast cancer (44/162, 27.2%), and were reported across other diagnoses (e.g., leukemia 48/207, 23.2%, and lymphoma 53/251, 21.1%) to varying extents.

We do acknowledge underreporting of social concerns in our cohort and will discuss the potential reasons in our discussion. Accordingly, reported *p*-values for the association of social concerns with diagnoses may not be accurate. In addition, we recognize the heterogeneous group of AYA categorized in the other category (*n* = 57, 5.1%), but the same analysis run with their exclusion did not impact the results and hence we have included them to give factual data. The expected cell size according to our analysis was ≥5, hence those with less than 5, should not be considered applicable to analysis (Table 3).

### Impact of time since diagnosis on concerns

Median time since diagnosis to PM AYA program consultation in the evaluable cohort of 943 AYA was 60 days (range 0–9599). The time since diagnosis was significantly associated with concerns in the following domains: fertility (*p* < 0.001), body image (*p* = 0.005), exercise (*p* = 0.010), diet (*p* = 0.028), and social support (*p* = 0.002). The highest proportion of patients with fertility concerns were in AYA who were assessed in the PM AYA program at diagnosis (0–30 days, 283/305, 92.8%), while the lowest proportion was among those assessed after 1 year of diagnosis (144/229, 62.9%). The highest proportion of body image concerns were reported between 91 and 365 days (36/167, 21.6%) and the lowest proportion were reported after 1 year of diagnosis (21/229, 9.2%).

The greatest proportion of exercise-related concerns was reported in AYA probably on active treatments postdiagnosis (31–90 days, 82/242, 33.9%). The remaining concerns (sexual health, coping, financial, and work/school) were not significantly associated with time since diagnosis.

## Discussion

The PM AYA program was established in response to a clear need for improvement in care of the AYA patient population. This program delivers individualized, timely, evidence-based, and age-appropriate care to address the unique physical and psychosocial needs of AYA with cancer. Through our analysis of concerns reported in the AYA program and their associations with age groups, diagnoses, and time since diagnosis, we present the most important learnings and ways to improve the program.

Our study revealed fertility as the most common concern AYA identified during the initial consultation across age groups, diagnoses, and time since diagnosis, reinforcing the key role fertility counseling plays in any successful AYA program. Our finding is consistent with other published reports from across cancer diagnoses.^17,18^ Furthermore, fertility concerns and unaddressed fertility information needs are associated with a reported lower quality of life,^19^ and in many situations, fertility preservation-related communication was delivered too late during treatment for AYA to make meaningful informed decisions.^20–22^

AYA have an expressed desire for information related to the impact of cancer on their fertility; and in fact, AYA have been found to experience psychosocial benefits from fertility counseling.^23,24^ AYA with cancer require support for myriad psychosocial needs; however, it is clear from our findings, and from the available literature, that fertility concerns must be prioritized in AYA oncology care. Our data support the hypothesis that if an AYA program is resource restricted, the most impactful intervention to prioritize is establishing pathways to address fertility concerns among the AYA.

Developing networks to fertility clinics and specialists, providing space and scope of fertility-related discussions through brochures, videos, infographics, and giving AYA access to these will serve a much-deserved need. We encourage others to freely use the Supplementary Data created by us—(PM AYA brochure, video)—for benefit of their patients.

The prominence of concerns related to school and work among our study population was also noteworthy, highlighting the need for more robust services that can aid AYA in this domain. This is consistent with Jones et al.'s finding that returning to work or school was the most reported practical concern among a cohort of 575 AYA cancer survivors surveyed across Canada.^25^

Important milestones such as graduating from secondary school, pursuing higher education, transitioning into the workforce, and gaining financial independence typically occur during AYA years. However, being diagnosed with cancer and undergoing treatment can affect AYA physically, cognitively, and psychologically.^9^ These effects can manifest as concentration problems, decreased energy and motivation, fatigue, changes in mood, chronic pain, and difficulties performing physical tasks,^26^ in turn affecting the ability to attend school or work, and thus to achieve their educational and career goals.^27^

It is clear from our findings and is abundantly previously documented that AYA programs could benefit from more robust services that involve vocational and occupational therapy expertise and can play a key role in the transition to healthy survivorship, fostering productive futures.^28–30^ This is especially true as the ability to RTW after cancer treatment has been associated with a better quality of life.^31^ Furthermore, the inability to RTW can result in significant financial losses at the level of the individual, family, and society.

Our study also found that concerns related to work or school differed among age groups, with the highest proportion being in the lowest age group (18–24 years). We could not find supporting evidence, but hypothesize that those in the older age groups may have more well-established careers and/or greater flexibility to take a temporary leave of absence to accommodate cancer treatment. In comparison, younger AYA may be at a stage in their lives where they are still in school or just beginning their careers, rendering a cancer diagnosis and subsequent treatments, a more significant interruption in their school or work.

Younger AYA particularly may experience challenges in this domain if they have had limited to no work experience or if they have short-term contract positions without insurance and/or benefit packages.^32^ Particular attention should be paid to offer younger AYA work and school counseling services and help. It behooves us to acknowledge this serious need for our patients and determine whether the resources to assist patients should be funded within or outside cancer programs (i.e., community-based occupational health). Nonetheless, patients need time and space to be able to voice these concerns and mechanisms in place for support.

In our study, the prevalence of concerns identified by AYA differed across diagnoses. Our study was not designed to determine the reasons for these differences; however, some of these differences are worth noting as they highlight the need for personalized approaches for AYA care. The greatest proportion of AYA who identified concerns with sexual health had a diagnosis of gynecological cancer. While a high prevalence of sexual dysfunction in patients with gynecological cancer has been well documented, the literature suggests an unmet need for information regarding sexual health in this population.^33–36^

What is unique about our study is that we report significant sexual health concerns across other diagnoses, indicating the need to address this issue in all AYA with cancer. In the oncology clinics, it might be busy to bring focus on such issues and hence the need for specialized AYA programs to discuss these important aspects of normal AYA lifestyle.

An additional finding that reinforces previous literature is the high proportion of body image concerns reported among AYA with breast cancer.^37,38^ The treatment of breast cancer may result in surgical breast removal and subsequent scarring, as well as hair loss and hyperpigmentation due to systemic therapies, all of which can potentially impact body image. Thus, interventions to maintain body image in AYA undergoing treatment for breast cancer should be prioritized in their treatment plan. Another noteworthy finding was the high proportion of work- and school-related concerns reported in patients diagnosed with sarcoma. While the impact of specific cancer diagnosis on RTW and school has not been extensively studied, this finding is in line with a recent study reporting that AYA patients with a diagnosis of hematological cancer or sarcoma were less likely to RTW.^39^

In a qualitative study conducted by Zambrano et al., sarcoma survivors identified several limitations on their capacity to perform tasks that they were able to complete with ease before their diagnosis.^27^ Some participants noted needing to take longer breaks or to work at a slower pace, whereas others had to find different jobs entirely.

Taken together, our findings reveal that the needs of AYA differ depending on their type of cancer diagnosis. As health care continues to move toward a patient-centered model, it is crucial to consider the impact of a particular diagnosis when developing a care plan.

The frequency of concerns related to social support was unexpectedly low in our AYA cohort, contrary to the research that suggests social support is an unmet need among AYA with cancer.^40^ Social support can be derived from various resources within one's social network. It is well established that support from both family and friends is instrumental for AYA throughout their cancer trajectory.^41^ However, AYA with cancer experience challenges in maintaining current friendships. In a qualitative study conducted by Breuer et al., AYA experienced a lack of support from friends as they reduced or completely stopped contact over their illness trajectory.^42^ Furthermore, a diagnosis of cancer and its treatment may impair their ability to stay connected with peers through social activities and work/school attendance. A unique type of support that is considered valuable to AYA is support from peer survivors.^43^

In fact, AYA placed a significantly higher value on interacting with other survivors than support from family and friends.^44^ This may be the case because survivors can provide advice on navigating challenges associated with cancer and the AYA can connect with them on a deeper level. Evidently, social support can be defined in a variety of ways. Perhaps, concerns related to social support were not appropriately defined and documented and hence not adequately recognized in our cohort, which we will endeavor to address in the future, especially regarding peer support mechanisms.

The PM AYA program accepts referrals across their cancer journey, at diagnosis, during treatment, and in survivorship. Our findings reveal that the prevalence of only certain health-related concerns was related to the time since patients received their diagnosis. Fertility concerns were reported predominantly in AYA patients who received a consultation within 30 days of their diagnosis. This highlights the critical need for discussions about fertility preservation to be initiated by oncologists immediately following diagnosis.

Concerns related to body image, exercise, and diet were most significantly reported by AYA who underwent consultation within 1 year of their diagnosis. We speculate that this period captures the time frame in which AYA are receiving their treatment. Therefore, it is important to provide AYA with access to multidisciplinary specialists such as dieticians, physical therapists, and social workers, while they undergo active treatment.

The strengths of our study are large sample size, across most common AYA cancers, and in demonstrating trends to prioritize particular AYA concerns. We acknowledge that our analysis is limited by being a single-center experience, noninclusion of important demographics like sex and ethnic background and their impact on AYA concerns, nonremoval of confounding effect of dependent variables, and further subgroup analysis to demonstrate significance of individual categories.

In summary, our analysis highlights the importance of any AYA program to prioritize addressing fertility concerns of AYA and second, supports for return to school/work, especially for those younger than 25 years. Implementing standard operating guidelines can streamline the process and improve access to information and resources for fertility preservation. Although this has been our focus since the beginning, we will continue to improve upon the processes to increase the scope and accessibility of this service.

To thoroughly address school and work concerns, it is important for AYA to have access to occupational therapists and vocational counselors. Access to such professionals helps AYA to successfully navigate an RTW or school, and in turn, improves their overall quality of life, impact on society, and overall well being. We plan to increase our engagement in this domain of care, realizing the need. Finally, moving toward diagnosis-driven individualistic holistic care models for AYA should be conceptualized and are the need of the future.

## Conclusions

We have successfully completed 7 years of a robust, comprehensive, service-based AYA program in a tertiary cancer care center and, in this study, present our learnings and observations. Concerns regarding fertility and work/school remain the most common among AYA. Age at diagnosis, diagnoses, and time since diagnosis meaningfully affect the journeys and concerns of AYA. We continue in our endeavor to improve upon from these learnings to deliver age-appropriate care to the AYA.

## Supplementary Material

Supplemental data
