# Supplementary material for: Looking Back to Move Forward: Lessons Learned from a Successful, Sustainable, Replicable Model of Adolescent and Young Adult Program of a Tertiary Cancer Care Center
Source: J Adolesc Young Adult Oncol. 2022 Apr 19;11(2):181–8. doi: 10.1089/jayao.2021.0156 (PMC9057888; doi:10.1089/jayao.2021.0156)
Supplement: Supplemental data [file Supp_Data.zip › JAYAO-2021-0156-Pitch_Suppl.Video.doc.docx]

**SUPPLEMENTARY INFORMATION**

SUPPLEMENTARY VIDEO S1. Video link to introduction of PMH AYA Program

[https://www.youtube.com/watch?v=--CEmCG4FXI&t=29s](https://urldefense.com/v3/__https:/www.youtube.com/watch?v=--CEmCG4FXI&t=29s__;!!CjcC7IQ!ZNjyu4rdEVFr_7KmwcVSe9rdVpv1cxePQr0_UYc4ueTo9sfxPsn-nN1h_BFa13ib$)

About the general introduction to the AYA program:

**SUPPLEMENTARY VIDEO S1.** White-Board Video created for introduction to the PM AYA program (introduction).

SUPPLEMENTARY VIDEO S2. Video link to Easy explanation on "Egg Preservation - An Option for people who have ovaries and are at risk for sub-fertility."

[https://www.youtube.com/watch?v=wB9lfKlYReY](https://urldefense.com/v3/__https:/www.youtube.com/watch?v=wB9lfKlYReY__;!!CjcC7IQ!ZNjyu4rdEVFr_7KmwcVSe9rdVpv1cxePQr0_UYc4ueTo9sfxPsn-nN1h_M2iDYCb$)

About the video on female fertility preservation procedure:

**SUPPLEMENTARY VIDEO S2.** White Board Video created by PM AYA program for simple explanation on Female Fertility preservation process.

**Legend 1** - Fertility Preservation information for women starting cancer treatment.

**Legend 2** - Fertility Preservation information for men starting cancer treatment.
