# Supplementary material for: Looking Back to Move Forward: Lessons Learned from a Successful, Sustainable, Replicable Model of Adolescent and Young Adult Program of a Tertiary Cancer Care Center
Source: J Adolesc Young Adult Oncol. 2022 Apr 19;11(2):181–8. doi: 10.1089/jayao.2021.0156 (PMC9057888; doi:10.1089/jayao.2021.0156)
Supplement: Supplemental data [file Supp_Data.zip › Fertility_Preservation_for_Men_Starting_Cancer_Treatment.pdf]

Fertility Preservation

For Men Starting Cancer Treatment

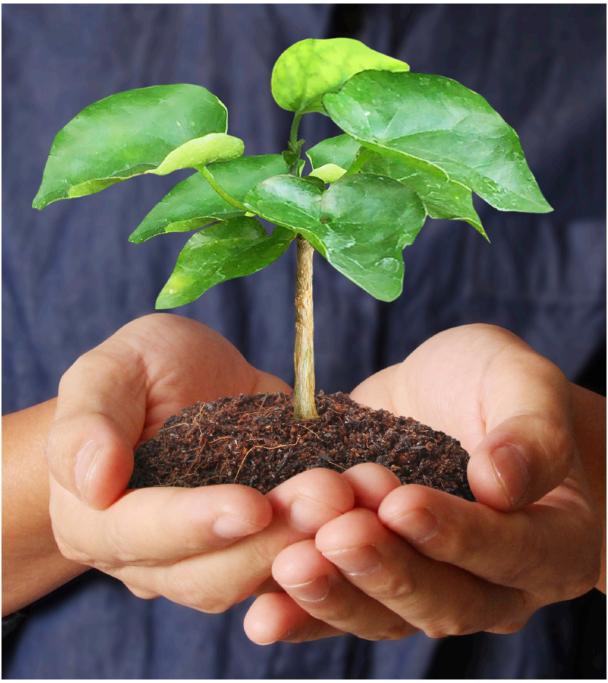

The ability to have children is often a concern for many men starting cancer treatment.

This pamphlet provides information on sperm banking, the most commonly used fertility preservation method for men.

UHN Princess Margaret Cancer Centre

Men begin to produce sperm once they reach puberty and will continue to do so over time as their testicles begin to produce testosterone. Sperm is contained in semen that is released during ejaculation through masturbation or sex.

How does cancer treatment affect my fertility?

Cancer treatment (chemotherapy, radiation, bone marrow transplant) can damage the quality of your sperm and can result in a low sperm count or loss of sperm production. The effects on your fertility will depend on the types of treatment you receive. However, because your cancer and treatment trajectory can be unpredictable, it is recommended to sperm bank prior to receiving any type of treatment. Although you may not want to have children now, sperm banking will give you the option of having your own children later on.

How can sperm banking preserve my fertility?

Sperm banking will store sperm that has not been exposed to treatment so it can be used in the future. It is important to continue storing your banked sperm until you decide to start a family.

When you are ready to have children, your stored sperm can be:

- Injected directly into a partner's uterus (intrauterine insemination)
- Fertilized with a partner's egg in a test tube to create an embryo (in vitro fertilization), which would then be placed into a partner's uterus

Success rates of fertility treatments are constantly evolving and can vary depending on the clinic. A fertility specialist can provide more specific information based on your case.

When should I sperm bank?

You need to discuss the possible risks with your oncology team and make a decision as soon as you can.

It is recommended that you sperm bank **before** starting treatment, because it is possible that after treatment you may no longer be producing any healthy sperm.

Many people decide to bank more than once as the sperm in the first sample can be unsuccessful in getting a partner pregnant. It is suggested to wait 2 days between each banking and abstain from ejaculation for 2 to 3 days before you bank sperm, if possible.

What happens if I need to start treatment right away?

Sperm banking can be arranged to be done quickly. Discuss this with your oncology team as soon as possible so they can refer you to a fertility clinic.

If you are unable to delay treatment, it is encouraged that you ask your oncology team or the Adolescent and Young Adult (AYA) Program about doing a semen analysis about 6 to 12 months after treatment is complete, and on a yearly basis. This test will assess the health of your sperm and will determine if your sperm count is returning to a normal range. If not, other options for family planning can be offered.

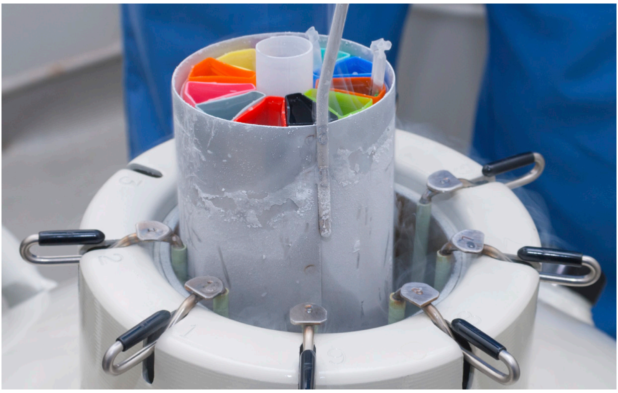

Where do I go for sperm banking?

Book an appointment with a fertility clinic through the AYA Program, or your oncology team. If you are unable to leave the hospital, you can still create a sperm sample within the privacy of your room. Your oncology team can arrange for it to be stored at a fertility clinic nearby.

A list of Canadian fertility clinics can be found through the **Cancer Knowledge Network:** www.cancerkn.com

What are the costs involved?

Government-funded fertility services are available to eligible patients. For more information about coverage under the Ontario Fertility Funding, visit www.ontario.ca/fertility

Type	Cost
Sperm Banking	About $350
Additional Sperm Sample	About $100
Yearly Storage Fee	$300
In Vitro Fertilization	Some OHIP coverage available but costs approximately $10,000
Intrauterine Insemination	Some OHIP coverage available

Private health insurance plans may cover some of these costs.

Fertile Future's Power of Hope Program (www.fertilefuture.ca) can provide financial help if you qualify. **Phone:** 1-877-HOPE-066

Can I still have sex even if I can no longer produce any healthy sperm?

Even if you are no longer producing sperm, you can still have sex. However, with some treatments and cancers, the nerves around the penis and testicles may be affected, making it difficult to have sex or carry an erection. Please ensure the use of contraception as a safety precaution to avoid pregnancy during cancer treatment. If you have concerns with your sexual function, please speak to a member of your healthcare team.

What happens at the fertility clinic?

PREPARATION
You will be taken into a private room

PRODUCTION
You will masturbate to produce semen that will be collected in a cup (magazines and videos are often provided)

EXAMINATION
Your sperm will be examined to determine its health and count number

STORAGE
Your sperm will be kept frozen until you decide to use or toss it

What if I have more questions or concerns?

Please ask any member of your healthcare team. If you feel more comfortable talking to a male staff member, this can be arranged.

In collaboration with Social Workers at Princess Margaret

For more information, please contact:

Adolescent and Young Adult Program

AYA Oncology Program
Princess Margaret Cancer Centre
Phone: **416-946-4501 ext. 5579**
Email: **aya@uhn.ca**
www.ayaprincessmargaret.ca

Fertility Clinics in Toronto

Mount Sinai Fertility
250 Dundas Street West, 7th Floor, Toronto, ON
Phone: 416-586-4748
www.mountsinaifertility.com

CReATe Fertility Centre
790 Bay Street, Suite 1100, Toronto, ON
Phone: 416-323-7727
www.createivf.com

Hannam Fertility
160 Bloor Street East, 15th Floor, Toronto, ON
Phone: 416-595-1521
www.hannamfertility.com

New Life Fertility
Multiple locations (see online)
Phone: 1-877-507-5433
www.newlifefertility.com

UHN Princess Margaret Cancer Centre

Version 2, December 2018 CSS# 73874
