# Supplementary material for: Looking Back to Move Forward: Lessons Learned from a Successful, Sustainable, Replicable Model of Adolescent and Young Adult Program of a Tertiary Cancer Care Center
Source: J Adolesc Young Adult Oncol. 2022 Apr 19;11(2):181–8. doi: 10.1089/jayao.2021.0156 (PMC9057888; doi:10.1089/jayao.2021.0156)
Supplement: Supplemental data [file Supp_Data.zip › Fertility_Preservation_for_Women_Starting_Cancer_Treatment.pdf]

Fertility Preservation

For Women Starting Cancer Treatment

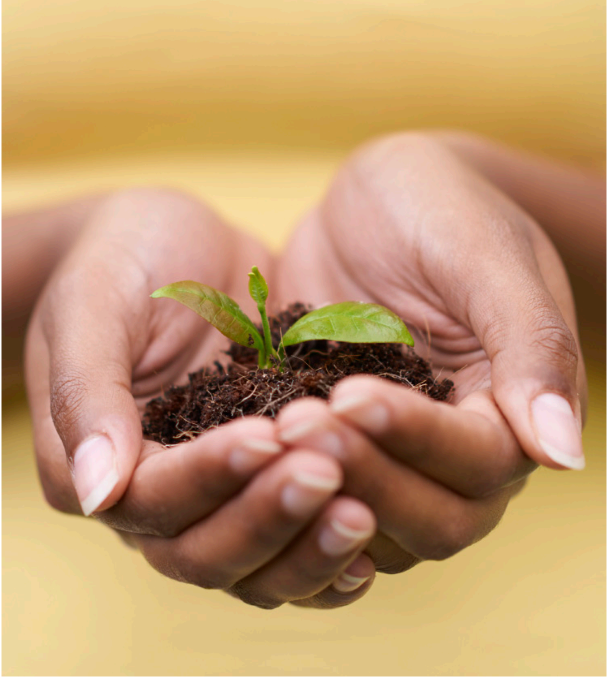

The ability to have children is often a concern for many women starting cancer treatment.

This pamphlet provides information on egg and embryo freezing, and how it may increase your chances of having a child one day.

UHN Princess Margaret Cancer Centre

If you are female, you are born with about 2 million eggs stored in your ovaries. The number of eggs in your ovaries will naturally decrease over time as you do not produce more eggs after you are born. When there are very few eggs left, you will enter menopause, which usually starts to occur around the age of 50.

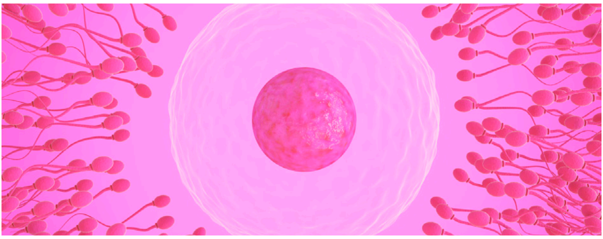

How does cancer treatment affect my fertility?

Cancer treatment (chemotherapy, radiation, bone marrow transplant) can sometimes cause you to lose your eggs earlier, which results in entering menopause at an earlier age. Your window of fertility can be reduced due to accelerated ovarian aging. This also is very dependent on your current age. In addition, the quality of your eggs can decrease when exposed to certain treatments.

As a result of cancer treatment, one of the following will occur:

1. No treatment-related effect on your eggs
2. Only a few healthy eggs left
3. No healthy eggs left – you will not be able to preserve any eggs after treatment

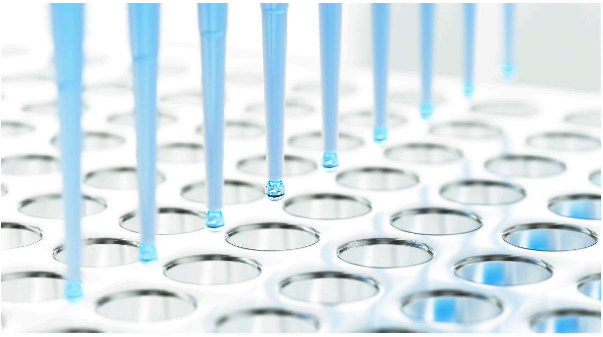

How can egg and embryo freezing help preserve my fertility?

Egg Freezing: This allows you to save your eggs so you can increase your chances of having your own biological baby in the future, even if your fertility is low.

Embryo Freezing: This allows you to fertilize your eggs with sperm (from a partner or donor) through in vitro fertilization to form an embryo (the first stage of a baby's growth) before freezing.

Embryo freezing gives you a better chance of having a child than egg freezing. The success rate of pregnancy for egg freezing is about 5 - 10% per egg, while embryo freezing is about 30 - 50% per embryo. The more eggs or embryos you freeze, and the younger you are, the higher your rate of success. Success rates of fertility treatments are constantly evolving and can vary depending on the clinic. A fertility specialist can provide more specific information based on your case.

When should I decide whether to freeze my eggs or embryos?

It is recommended that you decide **before** starting cancer treatment whether you will:

1. Freeze eggs/embryos now
2. Freeze eggs/embryos after you finish treatment
3. Not freeze eggs/embryos at all

The choice you make will depend on your age, type of cancer treatment, and urgency to start treatment. The process of preserving your eggs and embryos can take about 2 to 3 weeks. If you are unable to delay your treatment, egg or embryo freezing may not be a safe option for you.

You can ask the Adolescent and Young Adult (AYA) Program or your oncology team about doing an ovarian function assessment about 8 to 12 months after treatment to determine the number and quality of eggs remaining. If ovarian function is still intact, women under 43 may still have the opportunity to freeze eggs and embryos after treatment.

Where do I go to freeze my eggs or embryos?

Book an appointment with a fertility clinic through the AYA Program, or your oncology team.

A list of Canadian fertility clinics can be found through the **Cancer Knowledge Network:** www.cancerkn.com

What are the costs involved?

Government-funded fertility services are available to eligible patients. For more information about coverage under the Ontario Fertility Funding, visit www.ontario.ca/fertility

Type	Cost
Egg Freezing	1 cycle covered by OHIP if under 43 years (embryo freezing may have additional costs)
Fertility Medications	$2,000 to $5,000 (private insurance may cover some or all of this)
Annual Storage Fee	$300 to $500
In Vitro Fertilization	Some OHIP coverage is available

Private health insurance plans may cover some of the costs.

Fertile Future's Power of Hope Program (www.fertilefuture.ca) can provide financial help if you qualify. **Phone:** 1-877-HOPE-066

Can I carry a pregnancy even if I can't conceive naturally?

Many women who are no longer able to conceive after cancer treatment may still have the option of using their frozen eggs and embryos to carry a pregnancy. This becomes harder to do if you have had radiation to the pelvis or a bone marrow transplant. These treatments can damage the lining of your uterus where the baby is carried, or can affect the hormones needed to carry a pregnancy. You will need to speak to a fertility specialist about options regarding surrogacy.

What happens at the fertility clinic for egg and embryo preservation?

→ Egg Preservation → Embryo Preservation

PREPARATION
Go through blood tests and transvaginal ultrasound (probe inserted into vagina to look at ovaries)

STIMULATION
Hormone medications are given through a needle to stimulate your ovaries to produce eggs

SURGERY
Eggs will be collected through day surgery. Pain medications will be given as you will be awake during it

EXAMINATION
Eggs will be examined to make sure they are healthy

IN VITRO FERTILIZATION (IVF)
Eggs are fertilized with sperm in a test tube to form an embryo

STORAGE
Eggs and embryos will be safely kept frozen using liquid nitrogen

RECOVERY
When you are ready to conceive, your eggs or embryos will be thawed

IN VITRO FERTILIZATION (IVF)
Eggs are fertilized with sperm in a test tube to form an embryo

IMPLANTATION
An embryo will be placed inside your uterus (womb)

What if I have more questions or concerns?

Please ask any member of your healthcare team. If you feel more comfortable talking to a female staff member, this can be arranged. View our female fertility video on YouTube at https://youtu.be/wB9lfKlYReY

In collaboration with Social Workers at Princess Margaret

For more information, please contact:

Adolescent and Young Adult Program

AYA Oncology Program
Princess Margaret Cancer Centre
Phone: **416-946-4501 ext. 5579**
Email: **aya@uhn.ca**
www.ayaprincessmargaret.ca

Fertility Clinics in Toronto

Mount Sinai Fertility
250 Dundas Street West, 7th Floor, Toronto, ON
Phone: 416-586-4748
www.mountsinaifertility.com

CReATe Fertility Centre
790 Bay Street, Suite 1100, Toronto, ON
Phone: 416-323-7727
www.createivf.com

Hannam Fertility
160 Bloor Street East, 15th Floor, Toronto, ON
Phone: 416-595-1521
www.hannamfertility.com

New Life Fertility
Multiple locations (see online)
Phone: 1-877-507-5433
www.newlifefertility.com

UHN Princess Margaret Cancer Centre

Version 2, December 2018 CSS# 73874
